# Performance Assessment of a New Variable Stiffness Probing System for Micro-CMMs

**DOI:** 10.3390/s16040492

**Published:** 2016-04-08

**Authors:** Khalid Alblalaihid, Peter Kinnell, Simon Lawes, Dorian Desgaches, Richard Leach

**Affiliations:** 1 Manufacturing Metrology Team, Advanced Manufacturing Metrology Research Group, Faculty of Engineering, The University of Nottingham, Nottingham NG7 2RD, UK; eaxksa@nottingham.ac.uk (K.A.); S.Lawes@nottingham.ac.uk (S.L.); Richard.Leach@nottingham.ac.uk (R.L.); 2 EPSRC Centre for Innovative Manufacturing in Intelligent Automation, Wolfson School of Mechanical and Manufacturing Engineering, Loughborough University, Loughborough LE11 3UZ, UK; 3 École Nationale D’Ingéniurs De Saint-Étienne, Saint-Étienne 42023, France; dorian-desgaches@orange.fr

**Keywords:** probe sensor, stiffness modulation, micro-CMM

## Abstract

When designing micro-scale tactile probes, a design trade-off must be made between the stiffness and flexibility of the probing element. The probe must be flexible enough to ensure sensitive parts are not damaged during contact, but it must be stiff enough to overcome attractive surface forces, ensure it is not excessively fragile, easily damaged or sensitive to inertial loads. To address the need for a probing element that is both flexible and stiff, a novel micro-scale tactile probe has been designed and tested that makes use of an active suspension structure. The suspension structure is used to modulate the probe stiffness as required to ensure optimal stiffness conditions for each phase of the measurement process. In this paper, a novel control system is presented that monitors and controls stiffness, allowing two probe stiffness values (“stiff” and “flexible”) to be defined and switched between. During switching, the stylus tip undergoes a displacement of approximately 18 µm, however, the control system is able ensure a consistent flexible mode tip deflection to within 12 nm in the vertical axis. The overall uncertainty for three-dimensional displacement measurements using the probing system is estimated to be 58 nm, which demonstrates the potential of this innovative variable stiffness micro-scale probe system.

## 1. Introduction

Micro-scale coordinate measuring machines (micro-CMMs) make it possible to measure micro-scale features on parts that are small, intricate and fragile. Micro-CMMs can demonstrate very high accuracies, with volumetric uncertainty levels of a few hundred nanometres or less [1,2]. Micro-CMMs are operated in a similar fashion to conventional CMMs; however, probing on the micro-scale presents several significant challenges [3]. Micro-CMMs employ a high accuracy probing system, which is specially designed for measuring small and delicate parts. In particular, due to the small stylus tip size required by micro-scale probing systems, which can range in diameter from tens of micrometres to a few hundred micrometres [4], probe tip contact forces must be kept low (less than 1 mN [5]) to minimise surface damage [6].

To achieve low contact forces, micro-probes are designed to incorporate highly compliant suspension structures, onto which the stylus system can be mounted (for example, see [7,8,9]). When designing a low stiffness probe, consideration must also be given to the robustness, ease of manufacture, accuracy and dynamic response of the probing system. Surface attraction forces exerted on the stylus tip, when contact is made, can also cause significant issues [10]. If a probe is made too flexible, then the surface attraction forces can make it impossible to remove the stylus tip from a surface without causing serious damage to the probing system. Probe stiffness is, therefore, a difficult parameter to optimise. In some circumstances, it is advantageous to have a low stiffness, while in other cases, it would be better to have high stiffness.

In recent work, a variable stiffness probing system has been proposed to tackle the problem of optimising probe stiffness, and minimise the need for design compromise. The variable stiffness probing system makes it possible to modulate probe stiffness, so the optimum stiffness can be selected as required. Stiffness modulation is achieved through the use of a novel suspension structure that is combined with a set of piezoelectric actuators. The actuators are configured to apply compressive forces to the suspension structure, resulting in a reduction of stiffness. Controlling the magnitude of the compressive force, therefore, allows the probe stiffness to be controlled. A full description of the design and working principle behind the variable stiffness probing system is presented elsewhere [11,12].

The previous work illustrates the concept of variable stiffness, and demonstrates the potential performance in terms of stiffness modulation. In this paper, the results for a performance evaluation are presented for this new form of variable stiffness probing system. A brief description of the probing system and the associated fixtures is first presented. Then, to set the context for the experimental evaluation, the intended measurement strategy for the probe is defined. In addition, the novel control system used to set, monitor and control probe stiffness is discussed. The method used to complete a three-dimensional (3D) characterisation of the probe is then presented. Based on the characterisation results, a number of probing system errors are evaluated, including linearity, repeatability and long-term stability. To investigate the capability to modify stiffness during use, the probe is assessed under different compliance conditions, and following repeated switching between these states.

## 2. The Variable Stiffness Micro-Scale Probing System

**Figure 1:**
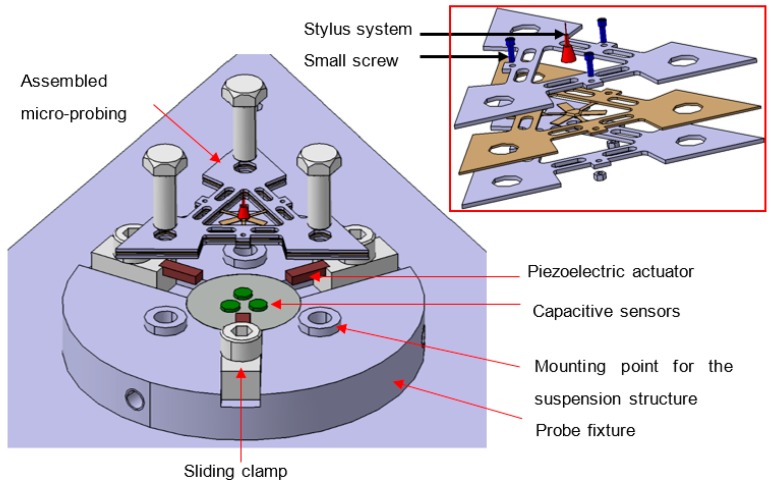
Schema of the assembly of the variable stiffness micro-probing system, with exploded view inset.

**Figure 2:**
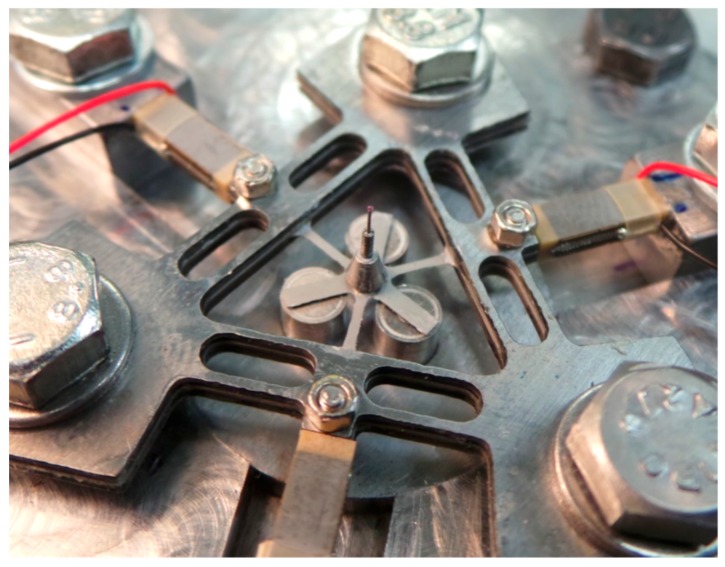
Assembled probing system, with stylus length 5.6 mm and 300 µm diameter ruby tip.

A schema of the variable stiffness micro-scale probing system is shown in Figure 1. The assembled suspension structure is the triangular-shaped object that is also shown in exploded diagram format. A stylus system with a 300 µm diameter spherical tip is mounted on to the assembled suspension structure. During probing, displacements of the stylus tip are measured as a function of suspension structure displacement; to measure this displacement, the suspension structure is mounted above three high precision capacitive displacement sensors. To allow the three sensors to be accurately positioned relative to the suspension structure, they are mounted within the probe fixture. The vertical position of the sensor within the probe fixture can be varied, so that the optimum working gap of approximately 100 µm can be set. To allow stiffness modulation of the suspension structure, three piezoelectric stack actuators (PICMA, P-882.11) with 8 µm maximum displacement are mounted to the probe fixture. To ensure that the actuators are positioned in intimate contact with the load application points on the suspension structure, a set of three sliding clamps are utilised. A photograph of the assembly of the variable stiffness probing system is shown in Figure 2, with the triangular suspension structure mounted to the probe fixture.

**Figure 3:**
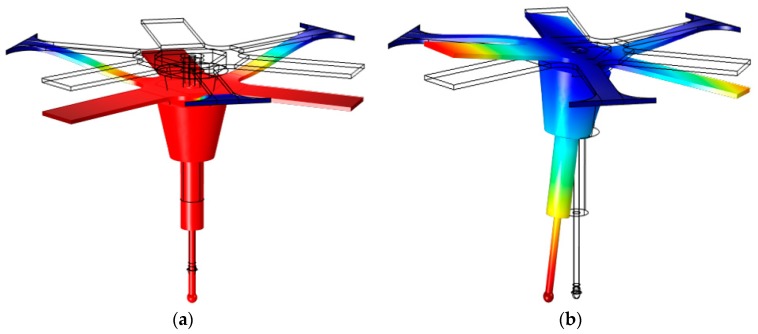
Shows mode shapes for zero applied compressive load: (**a**) the first mode (f = 1324 Hz), where the full micro-probe vibrates vertically; and (**b**) the second mode (f = 1700 Hz) where the centre of the micro-probe has a rotational motion.

The stiffness of the probing system can be modulated in two operating modes: “stiff” and “flexible”, which can be selected by switching the actuator force, supplied by the three actuators, between set-point values. To identify the expected vibration modes of the probing system, modes representative of vertical and lateral stiffness were estimated using finite element analysis of the full structure including the stylus system, and the selected mode shapes can be seen in Figure 3.

**Figure 4:**
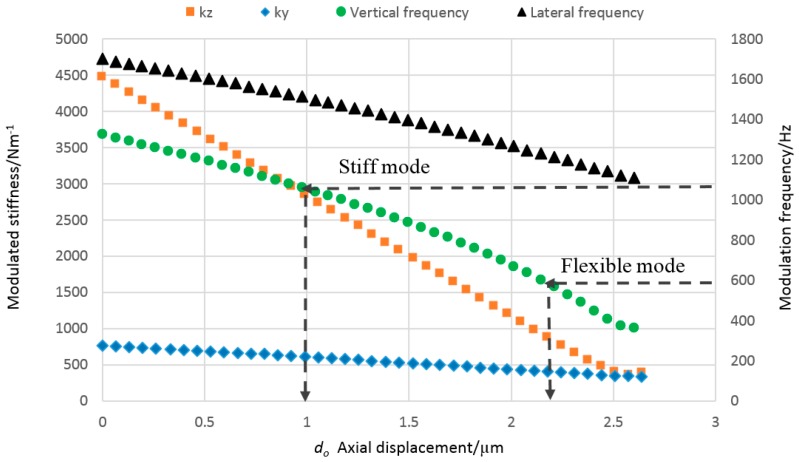
Simulated stiffness and frequency against the axial displacement, showing the stiff and the flexible mode of the probing system, as calculated by FEM.

To investigate the relationship between applied actuator load and probe stiffness, a finite element model (FEM) of the probe was developed. In the FEM, for simplicity, the effect of the actuator was modelled as a static displacement. For a range of applied actuator displacements, the stiffness and first two resonant modes of the probe were evaluated using a previously validated modelling process [11]. Figure 4 shows the simulated axial displacement (do), which is introduced by the piezoelectric actuator, plotted against the frequency and the stiffness of the probing system. The nominal working position of each mode is also shown on Figure 4, indicating the stiffness of the probe and the corresponding natural frequencies for each mode. The stiff mode is set at an actuator displacement of 1 µm, resulting in a modulation frequency in the vertical direction of 1057 Hz and a stiffness of approximately 2853 Nm^−1^ in the vertical direction, and around 610 Nm^−1^ in the lateral direction. For the flexible mode, the displacement is set at 2.2 µm and the frequency is 600 Hz; the vertical stiffness is 914 Nm^−1^ and lateral stiffness is 410 Nm^−1^, as illustrated in Figure 4.

### 2.1. Operating Sequence for the Variable Stiffness Probing System

The sequence of operation for a variable stiffness probing system consists of four steps. The first step is to drive the probing system to a position where the stylus tip is close to the measurement surface. During the first step, it is advantageous for the micro-CMM to move the probing system as fast as possible towards the target surface. Therefore, the probe will be in the stiff mode to prevent inertial loads, generated as the probing system is moved by the micro-CMM, from causing misleading deflections of the probe tip. The second step is to switch the probe to be in a flexible mode, so it is ready to make contact with the target surface. During this step, a potential difference is applied across the piezo-electric actuators to achieve the desired reduction in stiffness. To ensure that a repeatable stiffness value is reached, a feedback control loop is used (see Section 2.2). The third step is to carefully move the probing system towards the target surface, so that the stylus tip makes a controlled contact with the surface. During this step, the frequency-based control loop is switched off, so the probe operates in a purely static mode. The fourth step occurs after a measurement has been made, when the probe must be removed from the surface. During this step, the probe is switched back to the stiff mode, which makes it easier to develop sufficient force at the stylus tip to overcome attractive surface forces. The increased probe stiffness reduces the risk of overstraining the probe, which could result in measurement errors or damage to the probing system.

### 2.2. Control Strategy for the Variable Stiffness Probing System

**Figure 5:**
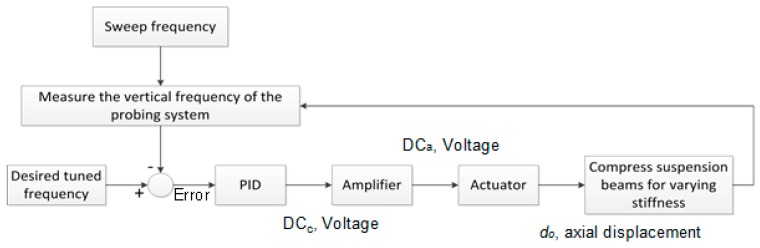
Block diagram of tuned stiffness for two modes (stiff and flexible). Note that DCc and DCa are the controlled voltage and amplified voltage, respectively.

The open-loop control capability of piezoelectric stack actuators is significantly limited due to hysteresis and creep effects [13,14]. Therefore, to use piezoelectric actuators in a high precision application requires the use of a closed-loop control system. For the variable stiffness probing system, a novel feedback control loop has been designed. The first modal frequency of the probe, which is a function of probe stiffness, is used as the control signal for the feedback loop. This is achieved by exciting the probe into resonance, and using the capacitive sensors to detect the resulting resonant frequency. The probe resonant frequency provides a control signal, which allows very repeatable probe stiffness values to be set. Figure 5 is the block diagram of the frequency-based proportional integral derivative (PID) control system. The feedback system also ensures that a constant probe tip position is set each time the probe is switched into flexible mode, which is important for ensuring accuracy and repeatability each time the probe is switched between the two modes.

To operate the flexible probing system in the two modes requires the use of two PID control loops. The two loops were implemented in LabVIEW software from National Instruments; Table 1 shows the specifications of the control system for tuning the frequency in both modes. In each case, the piezoelectric actuators were used to apply a superposition of a large static load combined with a small sinusoidal load: 0.3 V and 0.1 V for the stiff and flexible modes, respectively. The static load is applied to provide a constant level of compressive force to the suspension structure, which is used to control the probe stiffness. The sinusoidal load is used to excite, at the first mode of vibration of the probing system into resonance. To achieve this, a linear chirp signal is used to sweep the frequency from 1050 Hz to 1060 Hz for the stiff mode, and from 595 Hz to 605 Hz for the flexible mode. To analyse the resulting response of the suspension structure, a fast Fourier Transform (FFT) algorithm, also implemented using LabVIEW Real-time, was used.

## 3. Experimental Setup for the Probing System Characterisation

**Figure 6:**
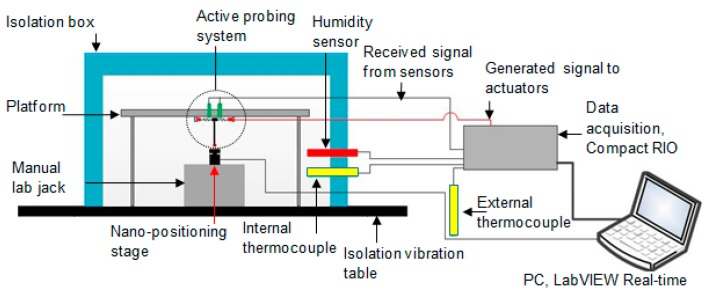
Schema of the setup for characterisation of the micro-probing system in z direction.

To provide a stable environment for testing the capability of the variable stiffness probing system, the experimental work was completed in a temperature controlled laboratory (20 ± 0.5 °C). An overview of the experimental setup is shown in Figure 6. The probing system was mounted on a three-legged platform placed on a pneumatic vibration isolation table; this minimised any disturbance due to vibration.

**Figure 7:**
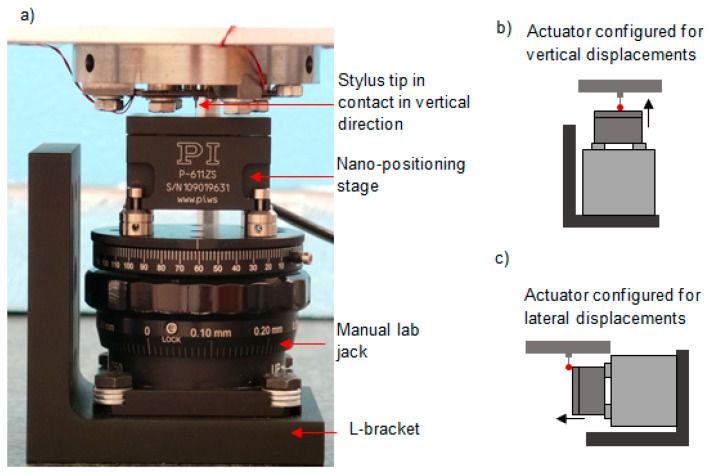
(**a**) An image of the experimental setup used for testing the micro-probe in the vertical direction; (**b**) actuator configured for vertical displacements; (**c**) actuator configured for lateral displacements.

To simulate the measurement process, a nano-positioning stage (Physik Instrumente, P-611.ZS) was used to displace the probe tip while the probe fixture remained static. The nano-positioning stage had a 100 µm maximum displacement, nonlinearity error of 0.1% and a repeatability of less than 10 nm [15]. In this work, the repeatability and linearity of the PI stage affects the determination of the sensor sensitivity matrix coefficients, the measurement of probe linearity and the measured repeatability of the probing system; as such the measurements presented should be regarded in relation to the quoted performance for the PI stage. Figure 7a is a photograph of the nano-positioning stage which was assembled with a manual lab jack to adjust the displacement between the stylus tip and the stage; in Figure 7a, the stage surface is perpendicular to the stylus, so vertical probe tip displacements can be applied (see Figure 7b). To characterise the performance of the probing system in the lateral directions, the nano-positioning stage could be repositioned using the L-bracket, which is shown in Figure 7a, so lateral displacements could be applied, as shown by the schemas in Figure 7c. A polished aluminium surface was attached to the head of the nano-positioning stage, so that a repeatable contact to the stylus tip could be made.

To provide additional environmental stability, the actuators and probing system were housed within a foam enclosure. This enclosure provided an insulating barrier to reduce thermal fluctuations, and reduce the impact of acoustic vibrations and air turbulence. Two thermocouples were used (see Figure 6) to measure the ambient temperature of the lab and the equipment setup inside the enclosure. A humidity sensor was also installed within the enclosure to account for the impact of humidity, which is known to affect the performance of the piezo-electric actuators [16] and capacitive sensors [17].

Signal synchronisation and excitation were achieved using National Instruments hardware (NI cRIO-9022) and LabVIEW FPGA (field programmable gate array). A lookup table was used to store waveforms for the linear chirp signal in the FPGA. A control system was designed to operate the probe using LabVIEW Real-Time. Coordination of the FPGA and Real-Time controller was achieved using Direct Memory Access, and the resulting control loop had a cycle time of approximately 51 ms. The maximum measuring rate of the capacitive sensor was 10 kHz, which is well above the maximum expected resonant frequency for the probing system (approximately 1 kHz).

## 4. Suspension Structure Performance Evaluation

Two kinds of experiments have been designed to investigate the performance of the micro-probing system. First, the behaviour of the probing system in the stiff mode, where the system can work as a normal micro-scale probing system, is considered. This initial investigation determines long-term stability, sensitivity of the probe in 3D and repeatability. Second, the probing system is investigated in the flexible mode, and the ability of the probe to switch between stiff and flexible modes is examined. For example, is the probing system able to repeatedly switch from stiff to flexible mode with no resultant off-set errors, and will the sensitivity of the probing system be changed as a result of switching between modes?

### 4.1. Environmental Long-Term Stability

**Figure 8:**
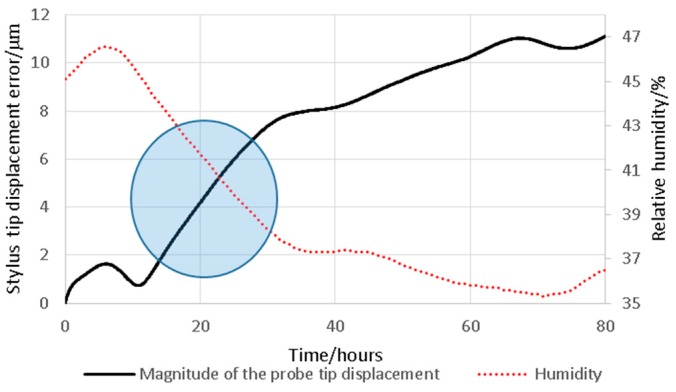
A plot of the magnitude of the stylus tip drift and relative humidity within the isolation enclosure over an eighty hour period. The steepest gradient area is indicated with an overlayed circle; in this region the rate of change of stylus tip position reaches a maximum of 6 nm per minute.

The stability of the probing system in the lab environment was assessed over a continuous eighty hour period. During this time, the probing system output, and the temperature and humidity in the isolation enclosure (see Figure 6) were recorded. Within three hours of introducing the isolation enclosure, the rate of change of temperature was reduced to 0.005 °C per hour for the duration of the eighty hour test. However, a more dramatic change was observed for the relative humidity over the same sample period, increasing from 35% to 47%. During this period, the probing system output was shown to drift by an amount equivalent to an 11 µm displacement of the stylus tip position. It can be seen in Figure 8, that there is an inverse correlation to stylus tip displacement error. As capacitive displacement sensors are known to be effected by the conductivity of the air gap [17], and conductivity closely relates to humidity, it would be reasonable to assume causation. The maximum observed rate of change of stylus tip displacement was shown to be 6 nm per minute, where the relative humidity was changed from 45% to 39% over sixteen hours. No attempt was made to compensate for this drift; however, to mitigate the impact of humidity, the time period of all subsequent tests was kept to a minimum. The time periods for each experiment are given with the detailed experimental description provided in the following sections.

### 4.2. Performance in Stiff Mode

**Figure 9:**
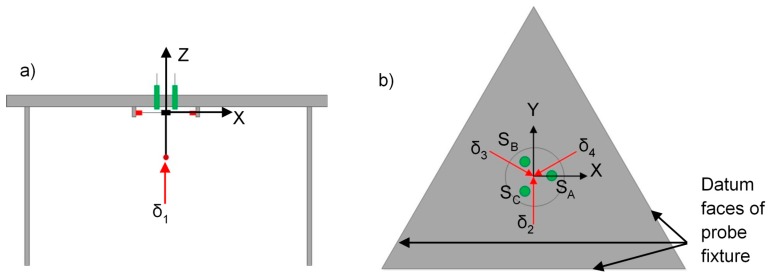
Schema showing (**a**) a front view of the platform that was used to calibrate the probing system in z axis; (**b**) a top view of the platform that indicate the position of three capacitive sensors and the calibration directions of the probing system in x and y axes.

In order to accurately measure the position of a probed surface, a set of coefficients are required that can relate the sensor measurements to the Cartesian position of the stylus tip. For the probing system presented, it is not convenient to directly observe Cartesian displacements, *i.e.*, displacement along the x, y and z axes, as shown in Figure 9. Instead, it is necessary to first relate sensor response to motion along the more convenient δ_1_, δ_2_, δ_3_ and δ_4_ displacement vectors described below, and then relate these to motion in a Cartesian frame (this is hereafter referred to as calibration). To calibrate the probing system in the stiff mode, the stylus tip was displaced over an incremental range using the nano-positioning stage. To minimise the effect of humidity-related drift, the calibration was conducted over a one minute period for each direction. To correctly align the nano-positioning stage for the three lateral displacements, the triangular body of the probe fixture was used to provide datum faces (see Figure 9b). To approach the probe tip, 100 nm incremental steps were carried out until contact was registered by the micro-probing system. Once contact had been made with the stylus tip, the output from the three capacitive sensors was recorded over a displacement range of 14 µm, with incremental steps of 2 µm.

**Figure 10:**
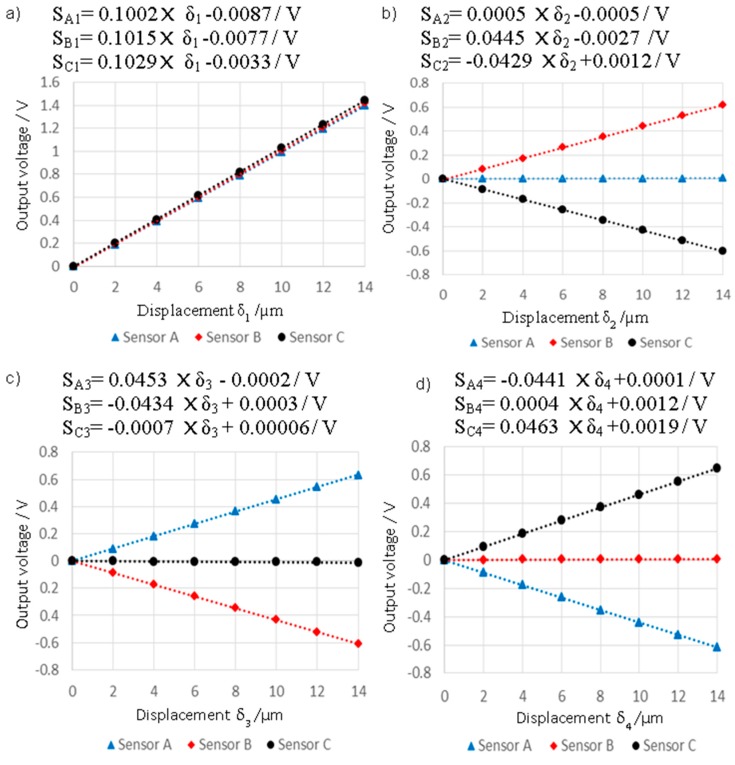
Output voltage from three capacitive sensors against the displacement of the probe tip in each of the four displacement ranges.

The output from the three capacitive sensors, for δ_1_, δ_2_, δ_3_ and δ_4_ displacement vectors, can be seen in Figure 10a–d, respectively. It is clear that all capacitive sensors read almost the same output voltage for displacements in the z direction, suggesting that the structure does not twist when displaced vertically. However, the displacement of the stylus tip in the y direction leads to rotation of the intermediate body about the x axis, where sensor B and C respond in opposite sign, but similar magnitude, and sensor A remains constant due to its positioned at the centre of rotation.

The data in Figure 10 was used to form a 3 × 4 matrix of sensitivity coefficients based on a least-squares linear fit. A sensitivity matrix can be approximated using the first-order Taylor series when the working range is small [18], see Equation (1)

(1) $$\left[\begin{array}{c} \Delta S_{A}\\ \Delta S_{B}\\ \Delta S_{C} \end{array}](V)=[\begin{array}{cccc} \frac{\partial S_{A1}}{\partial \delta_{1}} & \frac{\partial S_{A2}}{\partial \delta_{2}} & \frac{\partial S_{A3}}{\partial \delta_{3}} & \frac{\partial S_{A4}}{\partial \delta_{4}}\\ \frac{\partial S_{B1}}{\partial \delta_{1}} & \frac{\partial S_{B2}}{\partial \delta_{2}} & \frac{\partial S_{B3}}{\partial \delta_{3}} & \frac{\partial S_{B4}}{\partial \delta_{4}}\\ \frac{\partial S_{C1}}{\partial \delta_{1}} & \frac{\partial S_{C2}}{\partial \delta_{2}} & \frac{\partial S_{C3}}{\partial \delta_{3}} & \frac{\partial S_{C4}}{\partial \delta_{4}} \end{array}][\begin{array}{c} \delta_{1}\\ \delta_{2}\\ \delta_{3}\\ \delta_{4} \end{array}\right]$$

The coefficients in Equation (1) relate the displacement of the stylus tip in the four directions described (see Figure 9) to the three capacitive sensor voltage responses. Equation (2) [19] was used to give the inverse to the coefficients in Equation (1), thus

(2) $$\left[\begin{array}{cccc} 0 & 0 & \sqrt{3}/2 & -\sqrt{3}/2\\ 0 & 1 & -1/2 & -1/2\\ 1 & 0 & 0 & 0 \end{array}]=[\begin{array}{ccc} T_{XA} & T_{XB} & T_{XC}\\ T_{YA} & T_{YB} & T_{YC}\\ T_{ZA} & T_{ZB} & T_{ZC} \end{array}][\begin{array}{cccc} \frac{\partial S_{A1}}{\partial \delta_{1}} & \frac{\partial S_{A2}}{\partial \delta_{2}} & \frac{\partial S_{A3}}{\partial \delta_{3}} & \frac{\partial S_{A4}}{\partial \delta_{4}}\\ \frac{\partial S_{B1}}{\partial \delta_{1}} & \frac{\partial S_{B2}}{\partial \delta_{2}} & \frac{\partial S_{B3}}{\partial \delta_{3}} & \frac{\partial S_{B4}}{\partial \delta_{4}}\\ \frac{\partial S_{C1}}{\partial \delta_{1}} & \frac{\partial S_{C2}}{\partial \delta_{2}} & \frac{\partial S_{C3}}{\partial \delta_{3}} & \frac{\partial S_{C4}}{\partial \delta_{4}} \end{array}\right]$$

In Equations (1) and (2), the first subscript denotes the Cartesian axis they relate to, and the second subscript denotes the sensor. Finally, Equation (3) relates the sensor voltage directly to the displacement of the stylus tip with respect to x, y and z axes

(3) $$\left[\begin{array}{c} \delta_{x}\\ \delta_{y}\\ \delta_{z} \end{array}\right]=\left[\begin{array}{ccc} -12.9344 & 6.3118 & 6.3644\\ -0.2157 & 11.5794 & -11.2016\\ 3.3145 & 3.2608 & 3.2725 \end{array}\right]\left[\begin{array}{c} \Delta S_{A}\\ \Delta S_{B}\\ \Delta S_{C} \end{array}\right]$$

### 4.3. Repeatability in the Stiff Mode

**Figure 11:**
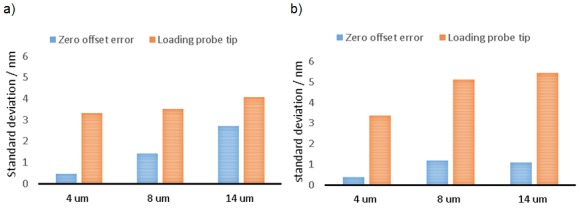
Bar charts showing repeatability in stiff mode for three nominal displacements (4 µm, 8 µm and 14 µm). (**a**) Shows results for the z direction; (**b**) shows results for the y direction.

It is important for accurate probe measurements that the stylus tip has a well-known and repeatable position before and after being displaced by contact. Therefore, the repeatability of the stylus tip initial position was evaluated for both lateral (y) and vertical (z) displacements (x direction data is omitted for brevity as it is fair to assume that it is equal to that in the y direction based on structural symmetry). For each displacement direction, the repeatable return to a datum position, henceforth called the “zero offset condition” was recorded for three nominal displacements of 4 µm, 8 µm and 14 µm. For each of the nominal displacements, the probe was displaced and then returned to the zero offset condition, with ten repeats being made at each displacement. Data is presented for both the return to zero offset, and the deviation between readings for the nominal displacements. Figure 11a,b show the result of repeatability in the z and y directions respectively. Note that the repeatability values were estimated by calculating the standard deviation of ten readings taken over ten repeated displacements of the probe tip followed by a return to the zero offset condition. It is clear that the suspension structure of the probing system has good repeatability, even for the larger, 14 µm displacement, where the zero offset was maintained at less than 3 nm vertically and 1 nm laterally. The zero offset error slightly increases as the nominal displacement for the z direction increases. The exact reason for the increase in zero offset error is not known; however, it is reasonable that as nominal displacement increases mechanical hysteresis and material related non-repeatability errors are also likely to increase.

### 4.4. Performance in Flexible Mode

**Figure 12:**
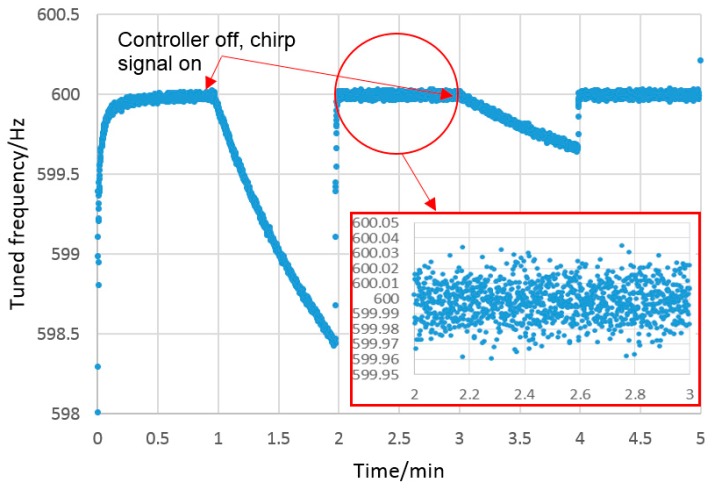
Frequency plot illustrating how actuator drift reduces following a series of actuator load cycles.

**Figure 13:**
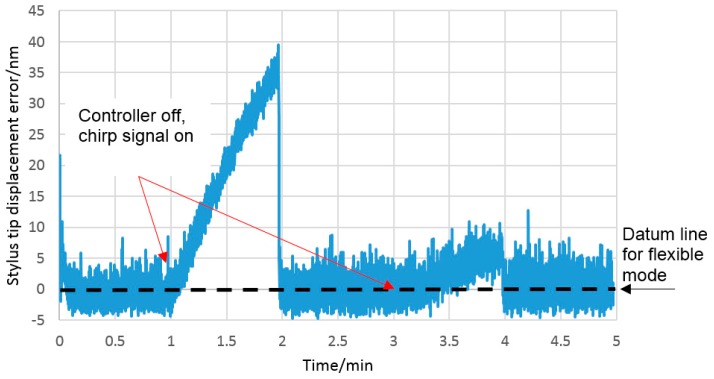
Frequency plot showing the magnitude of the stylus tip drift as a result of the impact of actuator creep at the flexible mode (600 Hz). This drift at the stylus tip and the tuned frequency in Figure 12 were measured at the same time.

As described in Section 2.2, stiffness control in the flexible mode is achieved using a frequency-based closed-loop control system, which requires the probing system to be vibrating. To avoid any potential errors due to probe tip vibration, the control system must be stopped prior to contact. This has the unwanted effect of allowing the piezo-electric actuator to drift without control. The amount of drift depends on the recent drive history of the actuators and creep can, therefore, be reduced by initialising the piezo-electric actuator using a set of load cycles prior to use [20]. The number of cycles needed to reduce the influence of the creep was investigated. The closed loop control was run for one minute to maintain the frequency at 600 Hz, and then the controller was switched to open loop to allow the drift to be measured. Figure 12 shows the result of an experiment where the vertical frequency was controlled to (599.997 ± 0.013) Hz and after one minute, the closed-loop control was switched off. The probe tip amplitude of the vertical mode of vibration was estimated from the AC voltage of the capacitive sensors and calculated to be approximately 2.4 µm. The sweeping chirp signal remains on to monitor the resonant frequency. It is clear that the piezoelectric actuators drift, which results in the first steep downward slope shown in Figure 12. Once the control system is restarted, the frequency rapidly returns to the 600 Hz set point. The test was repeated after a further minute, and it can be seen that the drift-related frequency change was greatly reduced. This trend was found to continue, and after a three minute period, drift was around 7 nm, as shown in Figure 13. Considering these results, ongoing tests of the probe in the flexible mode were conducted following an initial period of five minutes to reduce actuator creep.

Using the transfer function described in Equation (4) (see below), the impact of actuator creep on the stylus tip displacement was calculated. From Figure 13, it can be seen that after one and three minute periods, the actuator creep is around 35 nm and 7 nm at the stylus tip respectively. It is clear that the creep in three piezo-electric actuators was reduced as a result of repeating and recycling after a further minute.

**Figure 14:**
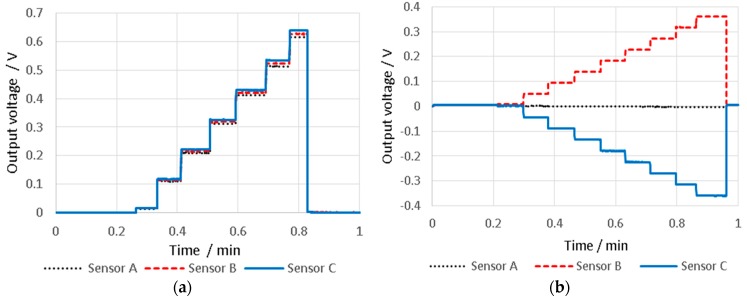
Output voltage from three capacitive sensors against the displacement of the probe tip in (**a**) z direction and (**b**) y direction, which are equal to 1 µm to generate probe calibration in vertical and lateral direction, respectively.

In order to investigate the sensitivity of the probing system in the flexible mode, it was calibrated using the same procedure as for the stiff mode. The position of the nano-positioning stage was maintained between the calibration of the stiff and flexible mode in each direction to minimise error. The calibration was completed within one minute, reasonably limiting drift to less than 6 nm, as described above. Figure 14a,b show the calibration time against the output voltage from the three capacitive sensors for displacement in the first and second directions respectively. The step height was 1 µm, and the datum point was taken as 100 nm beyond the first detected contact, to ensure a reliable contact between stylus and surface.

The transfer function resulting from the calibration of the four directions is given in Equation (4).

(4) $$\left[\begin{array}{c} \delta_{x}\\ \delta_{y}\\ \delta_{z} \end{array}\right]=\left[\begin{array}{ccc} -12.7610 & 6.1509 & 6.2700\\ -00.1463 & 11.3783 & -11.0249\\ 3.6676 & 3.2576 & 2.8760 \end{array}\right]\left[\begin{array}{c} \Delta S_{A}\\ \Delta S_{B}\\ \Delta S_{C} \end{array}\right]$$

**Figure 15:**
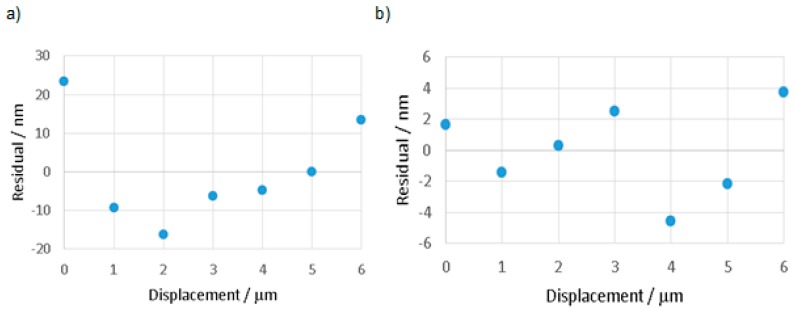
Plot shows the residual against fitted value, where the maximum nonlinearity is (**a**) 23 nm in z direction and (**b**) 4 nm in y direction for full range (6 µm).

In order to illustrate the linearity of the probe’s response to displacement, the residual is plotted in Figure 15a,b for the z and y directions respectively. It is clear that the maximum residuals in the z and y directions are approximately 23 nm and 4 nm, respectively.

To demonstrate that changing between modes does not significantly affect the calibration, a calibration was first performed in flexible mode, and then the probing system was set to stiff mode before returning to flexible mode for a repeat calibration. The coefficients generated from each calibration cycle are presented in in Table 2. There is a small change in the coefficients, approximately 0.4% and 0.9% in the z and y directions, respectively. To illustrate the effect of the repeated calibrations, the second set of calibration coefficients for direction 1 was applied to the first set of data, and a new residual error was calculated. For the z direction, the maximum residual errors were found to be 23 nm, and changed to 17 nm. In the y direction, the maximum residual errors were initially 4 nm and changed to 5 nm, which is an acceptably low change in residual error.

As well as determining the effect that switching from stiff to flexible mode has on sensitivity, it was also necessary to determine any unwanted stylus tip position offset error. This was determined with the knowledge that the load applied to the beams will result in a deflection of the stylus tip, which is most dominant in the z direction [12]. If the probing system was not able to return to the same initial zero offset when switched to flexible mode, it would directly impact the probing system error. The only option to reduce this error would be to recalibrate the probe each time the flexible mode was switched on, which would not be practical. As both natural frequency and displacement are functions of applied compressive force, it is possible to define displacement as a function of frequency; thus, for a given frequency there is an unambiguous corresponding displacement. The ability of the probing system to maintain a consistent zero position when set to flexible mode will therefore be limited by two factors. These factors are the ability of the control system to maintain a stable frequency, and the sensitivity of frequency to displacement change; for example, if the control system is able to maintain a perfect frequency with no error, then the displacement must also be perfectly controlled. However, if there is some error, then the degree to which this causes the stylus tip to be deflected will depend of the sensitivity of stylus tip deflection to frequency change; a low sensitivity will result in a low positional error and *vice versa*.

**Figure 16:**
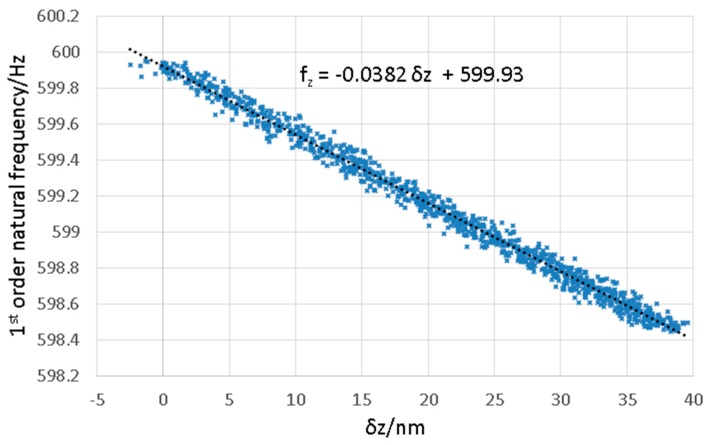
Plot showing the sensitivity of vertical frequency with respect to the displacement at the probe tip in the z direction.

To overcome this issue, the frequency-based probe stiffness control system (Section 2), was used. To determine the capability of the frequency-based probe stiffness control system to maintain stylus tip position, the relationship between stylus tip position (displacement in the z direction) and the natural frequency of the structure was determined; the results can be seen in Figure 16. This data was collected by setting the frequency control system to target 600 Hz, and then switching off the control system so only a steady state voltage is applied to the actuators. Under these conditions the actuators are free to creep under the influence of environmental conditions. This results in a steady increase in the applied force which results in the observed reduction in frequency from 600 to 598.4 Hz. While the frequency was reducing, the output from the capacitive sensors was monitored to allow the resulting displacement of the stylus tip to be measured.

The relationship is effectively linear over the small range of frequency deviations from the nominal 600 Hz control position. From a linear least-squares fit, the sensitivity of the deflection of the probe tip in the z direction as a function of tuned frequency is 26 nmHz^−1^. The control system is able to tune the frequency within a range of 0.075 Hz (see inset on Figure 12), so based on this the deflection, the probe tip can be controlled to within 2 nm in the z direction. Using frequency-based control, therefore, provides an effective method of controlling the flexible mode stiffness.

**Figure 17:**
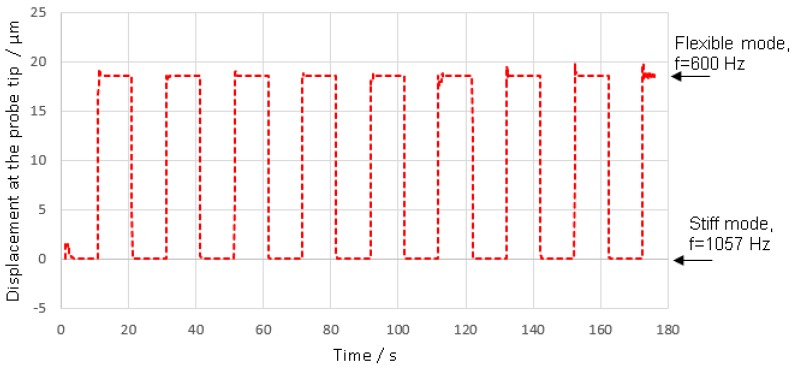
Plot showing control of the position of the probe tip in three dimensions between stiff and flexible mode using resonant frequency (the frequency of the stiff and flexible mode are 1057 Hz and 600 Hz, respectively).

The displacement that occurs between the “at rest” unloaded positions when making repeated switches between stiff to flexible control modes, henceforth referred to as switching repeatability, was determined. To measure the switching repeatability, the control mode was switched every ten seconds for a total of nine cycles, and the tip displacement monitored throughout. For this experiment the stylus tip was not constrained, ensuring true “at rest” position were measured. The results are shown in Figure 17. To obtain the data plotted in Figure 17, the DC voltage from the three capacitive sensors was recorded, in stiff and flexible mode, and then flexible and stiff calibrations were used to calculate the stylus tip positions. It is clear that the probe was deflected by a total magnitude of approximately 18 µm when transitioning to flexible mode.

The frequency according to Figure 17 can be controlled within 0.12 Hz (standard deviation). Therefore, the stiffness variability in the flexible mode can be calculated based on Equation (5); where $\sigma_{K}$, $\sigma_{f}$ and $m_{\text{eff}}$ denote the standard deviation of the vertical stiffness, the standard deviation of the tuned frequency and the effective mass. The effective mass in the vertical direction is estimated based on the FEA (65 mg), this gives a stiffness variability of 0.04 N/mm, which is 4.5% of the nominal value at 600 Hz.

(5) $$\sigma_{K}={\left(2\pi \sigma_{f}\right)}^{2}\;m_{eff}$$

Table 3 shows the repeatability for the performance of the control system switching between stiff and flexible mode. The maximum standard deviation is 12 nm in the z direction. This standard deviation is relatively high compared with the estimated capability to control the deflection within 2 nm. It is expected that, if longer periods than ten seconds were used to allow increased settling time for the control loop, the standard deviation would be reduced.

### 4.5. Repeatability of Flexible Mode Probing

The repeatability of the flexible mode was determined, in a similar manner to that in Section 4.3, by setting the probing system to flexible mode and taking a series of repeat measurements. The repeatability of the probing system was evaluated at zero displacement, and at a nominal displacement of 4 µm applied in the z and y directions. A total of ten repeat measurements were made over a one minute period. Table 4 shows the results for the repeatability test; the probe tip has a zero offset repeatability, after loading, with a standard deviation of 1 nm, and at the 4 µm applied displacement, the repeatability was 3 nm.

## 5. Discussion

The key novelty in the micro-probing system presented here, is the ability to switch between the stiff and flexible operating modes. To investigate this capability of the probing system, it was necessary to conduct several experiments to characterise its stability and repeatability in both the stiff and flexible operating modes. In addition, it was necessary to investigate the reproducibility of this performance following a switch of modes.

As a starting point, the stability of the probing system was characterised in both stiff and flexible modes. A small drift resulting from relative humidity variation was observed. This drift was expected due to the use of capacitive sensors within the probe, which are sensitive to changes in relative permittivity caused by humidity changes. To mitigate for this source of drift, an alternative sensing technology could be used, such as optical or piezo-resistive, both of which have been successfully demonstrated in micro-scale probing systems [3,21]. Alternatively, a compensation circuit using a reference capacitor or humidity sensor could be used to account for the drift. In this work, no compensation for the drift was made; instead all experimental work was conducted in a controlled laboratory environment, which limited humidity-related drift to less than 6 nm per minute (see Figure 8). A second source of drift, resulting from the piezo-actuators used in the probe, was also observed. To minimise actuator-related drift in future work, it is suggested that piezo-actuators with integral strain gauges should be used. These are commonly used in high precision nano-positioning stages, and can effectively eliminate actuator drift. In this work, standard actuators were used, and a period of actuator cycling was conducted to reduce actuator related drift to 5 nm per minute (see Figure 13). To further reduce the influence of both forms of systematic drift, all experimental procedures were conducted over as short a period of time as possible, typically one minute or less.

As it is intended that measurements will be made in the flexible mode, the performance of the probing system in this mode was examined from three perspectives. First, the maximum linearity error of the probing system was found to be 24 nm, over a full working range 6 µm, as presented in Figure 15. This value represents the magnitude of the stylus tip displacement, and is based on the assumption that the linearity error in the y direction is equal to linearity error in x direction. Second, the variation in stylus tip zero offset position, following excursions from flexible to stiff mode and then returning, was found to have a standard deviation of 12 nm (see Table 3). Third, while in the flexible mode, the probing system was able to measure a nominal 4 µm displacement with a standard deviation of 5 nm (determined by taking the magnitude of the z y and x values), the values for z and y are indicated in Table 4; again assuming that the repeatability in the y direction is equal to that in the x direction.

In addition to the probing system performance, the performance of the PI precision stage must also be considered. The precision stage used was quoted, by the manufacturer, to have 0.1% nonlinearity error and 10 nm repeatability. As the probing system was calibrated over a 6 µm range the expected nonlinearity error over this range is 6 nm. The repeatability of the PI stage was quoted by the manufacturer to be better than 10 nm; assuming the stage repeatability is uniformly distributed then the expected standard deviation of stage position can be estimate to be 6 nm.

Combining stage errors with the probing system linearity error, the stylus tip zero offset position error and the probing system measurement repeatability, gives an estimate of a combined uncertainty for the probing system or of 29 nm. The dominant uncertainty comes from the linearity error of the probing system. The expanded uncertainty is 58 nm (*k* = 2). This validates the feasibility of variable stiffness probing systems; showing the ability to switch between stiff and flexible modes with minimal impact on probing system performance.

While changing stiffness causes minor repeatability errors, it must be noted that as a result of switching, the stylus tip undergoes a displacement of approximately 18 um, which may cause issues for future application of the probing system. This displacement results from the tendency of the suspension structure to bend under the influence of uneven compressive load. The displacement at the probe tip is equal to −13.78 µm, 4.36 µm and 12.51 µm in x, y and z direction. The deflection of the stylus tip by this amount could cause large contact forces to occur when the probe is changed from flexible to stiff mode. To accommodate this displacement, three displacement sensors could be integrated to make sure three displacements are equal from each actuator, which would avoid the lateral deflection of probe tip during the translation between modes. Then to account for the motion in the vertical direction, the probe tip could be manipulated by the CMM in synchronisation with switching of the mode. This would limit the contact force generated by the probing system. Even if the displacement sensors were not applied, and a complex displacement in three directions was observed, the CMM could still be controlled in synchronisation with the probing system such that as the stiffness is changed the CMM retracts the stylus tip to compensate for any resulting deflection. This compensation procedure would be learned as part of the calibration routine for the probe, by slowly switching stiffness and monitoring the stylus tip deflection as a function of actuator voltage; the learned compensation displacement path would be applied by the CMM during stiffness mode transitions.

## 6. Outlook and Future Work

In this paper, the performance of the first variable stiffness micro-CMM probing system has been characterised. The evaluation was based on a large body of experimental data, that the interested reader is able to access via an online database [8]. Using this innovative probing technique allows the stiffness of the suspension structure to be modulated during probing; avoiding practical issues caused by the antagonism between the stiffness requirements of the probing system at different phases of a measurement.

A control system, based on measuring resonant frequency, has been developed and its ability to tune the stiffness in both stiff and flexible modes was demonstrated. When actuated from one mode to another, the suspension geometry is altered, and the stylus tip moves, but the rest position for both modes is shown to be predictable and repeatable to within 12 nm between the stiff and flexible mode in the z direction.

A practical limitation of the probing system is that it takes approximately twenty seconds to reach a steady state when the controller switches to flexible mode, as illustrated in Figure 13. Further research will be carried out to improve the speed of the probe; considering alternative geometric configurations and actuation mechanisms to reduce creep, humidity-related actuator drift and switching time.

The optimum stiffness for the probe would be zero stiffness for contacting and infinite for removal. In the paper we present a probe that has the ability to modulate frequency, and prove that it is possible to dynamically modulate stiffness during probing. However, more work is now needed to further increase the size of the stiffness change that is possible.

## Figures and Tables

**Table 1:** Specification of the control system used to tune the frequency in both modes.

Mode	Time Period/ms	DC Voltage Range/V	AC Voltage/V	Tuned Frequency/Hz	Sweeping Frequency Range/Hz
Stiff	51	5–10	0.3	1057	1050–1060
Flexible	51	41–55	0.1	600	595–605

**Table 2:** Sensitivity coefficients of the three capacitive sensors at the probe tip for displacement in z(δ_1_) and y(δ_2_) directions, before and after switching between flexible and stiff operating mode.

Item	Calibration Coefficients for Direction 1 (mV·µm^−1^)		Calibration Coefficients for Direction 2 (mV·µm^−1^)	
	First trial	Second trial	First trial	Second trial
Sensor A	$\frac{\partial S_{A1}}{\partial \delta_{1}}=100.5$	$\frac{\partial S_{A1}}{\partial \delta_{1}}=100.7$	$\frac{\partial S_{A2}}{\partial \delta_{2}}=0.6$	$\frac{\partial S_{A2}}{\partial \delta_{2}}=1.1$
Sensor B	$\frac{\partial S_{B1}}{\partial \delta_{1}}=101.9$	$\frac{\partial S_{B1}}{\partial \delta_{1}}=101.7$	$\frac{\partial S_{B2}}{\partial \delta_{2}}=44.6$	$\frac{\partial S_{B2}}{\partial \delta_{2}}=44.2$
Sensor C	$\frac{\partial S_{C1}}{\partial \delta_{1}}=103.9$	$\frac{\partial S_{C1}}{\partial \delta_{1}}=103.5$	$\frac{\partial S_{C2}}{\partial \delta_{2}}=45.1$	$\frac{\partial S_{C2}}{\partial \delta_{2}}=45.5$

**Table 3:** Stylus tip positional repeatability following repeated stiffness mode switching, calculated based on a series of eight repeated switches from flexible mode to stiff mode and then back to flexible mode, as illustrated in Figure 17.

Axis	*x*		*y*		*z*	
Mode	Stiff	Flexible	Stiff	Flexible	Stiff	Flexible
Standard deviation/nm	3	2	3	1	6	12

**Table 4:** Repeatability of the flexible mode for the x and y directions. The measurement was repeated ten times for each displacement (nominal displacement is 4 µm).

Axis	*y*		*z*	
Type of error	Zero off-set position	Nominal 4 µm displacement position	Zero off-set position	Nominal 4 µm displacement position
Standard deviation/nm	1	3	2	3
